# Pediatric Cancer in Northern Tanzania: Evaluation of Diagnosis, Treatment, and Outcomes

**DOI:** 10.1200/JGO.2016.009027

**Published:** 2017-06-09

**Authors:** Kristin Schroeder, Anthony Saxton, Jessica McDade, Christina Chao, Nestory Masalu, Colin Chao, Daniel S. Wechsler, Beda Likonda, Nelson Chao

**Affiliations:** **Kristin Schroeder**, **Anthony Saxton**, **Christina Chao**, **Daniel S. Wechsler**, and **Nelson Chao**, Duke University, Durham, NC; **Jessica McDade**, Seattle Children’s, Seattle, WA; **Nestory Masalu** and **Beda** **Likonda**, Bugando Medical Centre, Mwanza, Tanzania; and **Colin Chao**, Eastern Virginia Medical School, Norfolk, VA.

## Abstract

**Purpose:**

The majority of new diagnoses of pediatric cancer are made in resource-poor countries, where survival rates range from 5% to 25% compared with 80% in high-resource countries. Multiple factors, including diagnostic and treatment capacities and complex socioeconomic factors, contribute to this variation. This study evaluated the available resources and outcomes for pediatric patients with cancer at the first oncology treatment center in northern Tanzania.

**Methods:**

Qualitative interviews were completed from July to August 2015 to determine available staff, hospital, diagnostic, treatment, and supportive care resources. A retrospective review of hospital admissions and clinic visits from January 2010 to August 2014 was completed. A total of 298 patients were identified, and data from 182 patient files were included in this review.

**Results:**

Diagnostic, treatment, and supportive capacities are limited for pediatric cancer care. The most common diagnoses were Burkitt lymphoma (n = 32), other non-Hodgkin lymphoma (n = 26), and Wilms tumor (n = 25). A total of 40% of patients (n = 72) abandoned care. There was a 20% 2-year event-free survival rate, which was significantly affected by patient age, method of diagnosis, and year of diagnosis.

**Conclusion:**

To our knowledge, this is the first review of pediatric cancer outcomes in northern Tanzania. The study identified areas for future development to improve pediatric cancer outcomes, which included strengthening of training and diagnostic capacities, development of registries and research databases, and the need for additional research to reduce treatment abandonment.

## INTRODUCTION

An estimated 250,000 children worldwide are diagnosed with cancer every year, and the majority of diagnoses occur in low- and middle-income countries (LMICs).^1^ Although outcomes for childhood cancer have improved dramatically—survival rates are greater than 80% in high-income countries—rates in LMICs remain distressingly low, at 5% to 25%.^2,3^ Multiple factors, including the availability of trained personnel, access to tertiary health centers, limited treatment availability, cost of care, late stage of presentation, and treatment abandonment, contribute to the variation in outcomes in LMICs.^4,5^

Tanzania is a low-income country in east Africa, and an estimated 46% of Tanzanians live below the poverty line of $1.90 per day.^6^ Tanzania has an estimated population of greater than 53 million, and 45% of the population is younger than 15 years of age.^6^ There is no national cancer registry, so the population-based pediatric cancer incidence is unknown, but it is estimated at 134 occurrences per million.^3^ Historical published data on pediatric cancer distribution and outcomes in Tanzania are limited to a single site evaluation at the national cancer center in the eastern region of the country in Dar es Salaam, which served as the only cancer center in the country for many years. The historical reported pediatric cancer outcomes were poor: the overall survival rate was less than 20% in 2005.^3,7-10^ Since 2005, standardized treatment regimens, pediatric oncology training, and resource development at the national cancer center have led to significant improvement in overall survival rates, but access to care and presentation delays remain a challenge.^7,11^

To improve outcomes and increase access to cancer care, a second cancer center was established at Bugando Medical Centre (BMC) in 2010, and this center serves a catchment area of greater than 15 million people in the Lake Zone of northern Tanzania. Previous studies in adult patients with cancer have found variations in cancer incidence between eastern and northern Tanzania, which suggests that the distribution of pediatric patients in northern Tanzania may vary from that of the center in Dar es Salaam.^12^ In addition, determinations of the regional resources and current outcomes for pediatric cancer at BMC are needed to establish baseline information about disease burden and highlight areas for targeted interventions to provide the same quality treatment and improved outcomes across Tanzania.

A recent clinical and research collaboration with the Duke University Global Cancer Program and BMC has focused on improvements to the capacity of care for pediatric cancer patients in northern Tanzania. Through structured staff interviews and a retrospective chart review, we evaluated the treatment capacity, distribution of disease, and historical pediatric cancer outcomes. To guide future intervention planning, we also examined factors that may influence outcomes, including demographics, disease location, and treatment abandonment.

## METHODS

### Resource Availability

Qualitative interviews were conducted by K.S. from July to August 2015 to determine available staff, hospital, diagnostic, treatment, and supportive care resources. Ten key stakeholders were identified on the basis of their direct involvement in pediatric cancer care. These stakeholders were one oncologist, one radiation oncologist, two oncology staff physicians, one social worker, three nurses, one clinical pharmacist, and one pathologist. Interview questions were specialty directed to ensure accuracy. For example, the interviewed senior nurses provided information about nursing ratios. Data on determinants of staff resources included the availability of subspecialty physicians, social workers, hospital residents, pharmacists, and nurses. Data on determinants of hospital resources included the number of available pediatric oncology beds, nurse-to-patient ratio, diagnostic capabilities, and available treatment and supportive care resources. Data on diagnostic capability included the availability of histologic evaluation, bone marrow assessment, and radiology services and the availability of trained radiologists and pathologists to interpret the results. Treatment resources included the availability of radiation, pediatric surgeons, and essential chemotherapy as outlined according to WHO standards. Supportive therapy included the availability of blood products for transfusion, standard laboratory studies, pain medication, and antibiotics.

### Pediatric Oncology Distribution and Outcomes

A retrospective review of recorded hospital admissions and clinic visits from January 2010 to August 2014 was completed. We identified 298 patients, and data from 185 patient files were available for review. Three additional files were excluded because no malignancy was reported. Data collected for the remaining 182 patients included demographics (age, sex), clinical symptoms and duration at presentation, diagnostic modality (laboratory, radiology, histology), and treatment received. Survival outcome was classified as alive, deceased, or unknown. When outcome data were not specified in the file, active follow-up was attempted through interviews with the oncology social work team or through direct family contact, when phone numbers were available. Patients whose survival outcomes could not be confirmed were classified as unknown. All patients were additionally partitioned into three levels of treatment status: completion of therapy, abandonment of therapy, or incompletion of therapy because of reasons other than abandonment of care. Abandonment of therapy was defined as the inability to initiate or complete medically indicated curative therapy, and it was classified as an event in the event-free survival estimate.^13^

### Statistical Analysis

Descriptive statistical analysis was performed to determine the distribution of diagnoses and treatment by sex, age, and year of diagnosis. Event-free survival was estimated with the Kaplan-Meier method. Pearson χ^2^ tests were used to assess the relationship between patient variables and survival outcomes. Patients with unknown outcomes were excluded. Statistical analyses were performed with STATA v14.1 (StataCorp, College Station, TX). Geospatial analyses were performed with Tableau v9.3.1 (Tableau Software, Seattle, WA).

### Ethical Considerations

The study was approved by the Catholic University of Health and Allied Sciences/BMC Research Ethical Committee (Mwanza, Tanzania) and the National Institute for Medical Research—Lake Zone Medical Research Coordinating Committee (Mwanza, Tanzania). The study qualified for exemption (per 45CFR46.101(b)) by the Duke University Institutional Review Board (Durham, NC).

## RESULTS

### Resource Availability

Available resources for pediatric cancer care are listed in Table 1. The staff included both a fellowship-trained adult medical oncologist and a radiation oncologist, but the staff resources were limited by the lack of a trained pediatric oncologist. Initially, there were seven nurses who received additional oncology training. However, because of reallocation, only three remained. Diagnostic capacity was limited to basic radiologic services, which included x-ray and ultrasound. Although BMC does have two general pathologists, they are the only pathologists in the Lake Zone, and they receive samples from all district hospitals for processing. As such, there was often a 1-month delay in the return of histologic analysis of samples. Diagnoses of hematologic malignancies were limited by the lack of hematopathology training and flow cytometry.

**Table 1:**
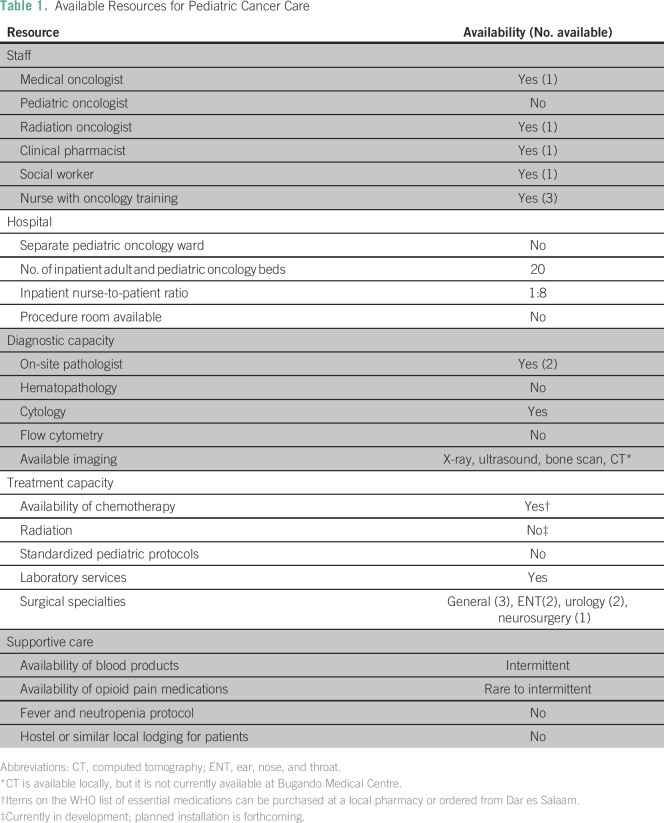
Available Resources for Pediatric Cancer Care

Although resources were limited, all of the WHO essential chemotherapies were available for purchase by the families through the local pharmacies. Basic supportive care, which included blood products and antibiotics, also were available intermittently on-site.

### Patient Characteristics

**Fig 1:**
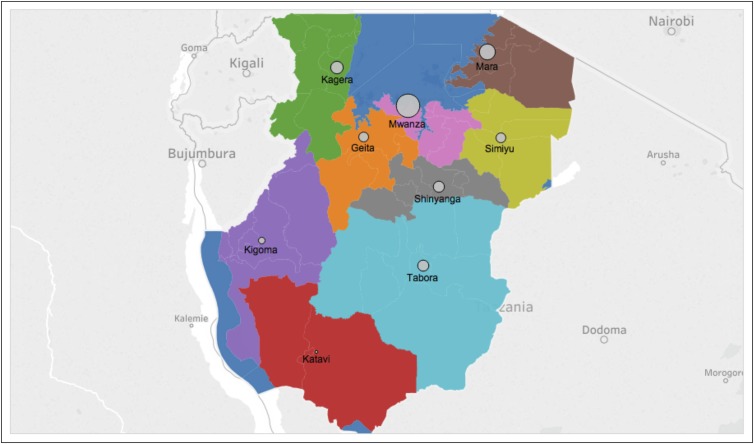
Patient distribution from the Tanzanian Lake Zone. Circle size is proportional to the number of patients who presented to Bugando Medical Centre from each region.

Forty-one percent (n = 74) of the 182 patients were girls, and the average age was 7 years (range, 1 month to 18 years). Patients traveled an average distance of 187 km (116 miles) to the clinic, and the estimated average travel time was 4.5 hours. All regions within the Lake Zone were represented; 39% were from the Mwanza region, where BMC is located (Fig 1).

### Diagnosis

The average time to diagnosis was 49.1 days. Histopathology diagnosis was made in 42% of patients (n = 77), and the remainder used imaging or clinical presentation for diagnosis. The most common recorded diagnoses were Burkitt lymphoma (n = 32); non-Hodgkin and other lymphoma, not otherwise specified (NOS; n =26); Wilms tumor (n = 25); acute leukemia (ie, acute lymphoblastic leukemia; acute myeloid leukemia; and leukemia, NOS; n = 24); and retinoblastoma (n = 20; Table 2).

**Table 2:**
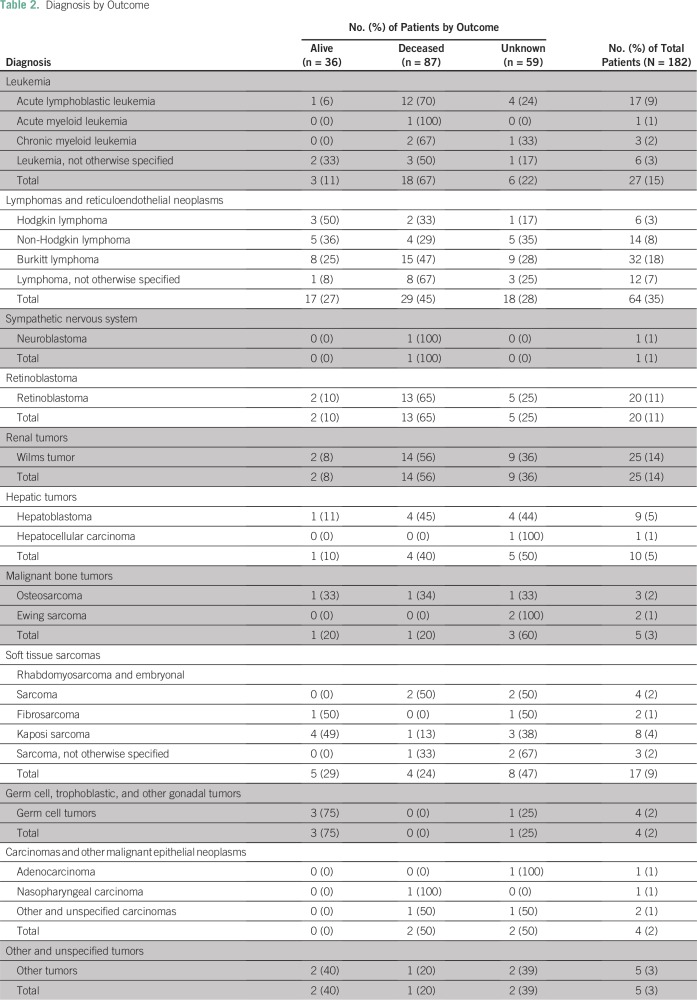
Diagnosis by Outcome

### Outcomes

**Fig 2:**
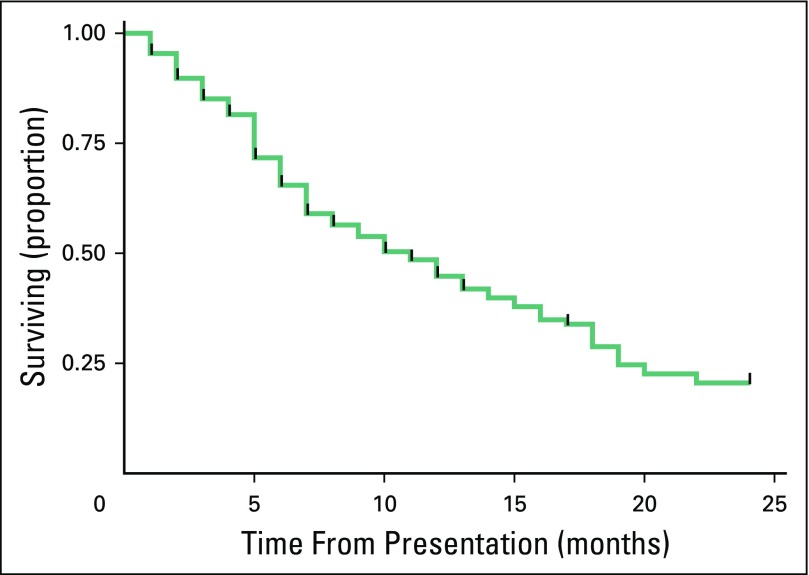
Two-year event-free survival for children with all types of cancer who were diagnosed at Bugando Medical Centre from 2010 to 2014 (n = 182).

**Fig 3:**
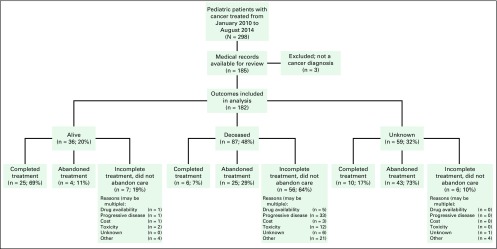
Treatment status by survival outcome for children with all types of cancer who were diagnosed at Bugando Medical Centre from 2010 to 2014 (n = 182).

There was a 2-year event-free survival rate of 20% (n = 36; Fig 2). A total of 48% of patients (n = 87) died, and 32% of patients (n = 59) had unknown outcome status (Fig 3). Of the 87 recorded deaths, 45% (n = 39) were due to treatment-associated toxicity, 30% (n = 26) resulted from disease progression, and 25% (n = 22) occurred after treatment abandonment because of an unknown cause. A statistically significant relationship was identified between patient survival outcomes and age (*P* < .01); the highest percentage of survival (40%) occurred in patients age 12 years or older (Table 3). Patients who had a histologic diagnosis had an increased survival rate compared with patients who had a clinical diagnosis (29% *v* 13%; *P* = .02). A statistically significant relationship also was identified between patient outcomes and year of diagnosis (*P* = .03); the highest 2-year survival rates (26%) occurred with diagnoses made in 2014. There was no statistically significant relationship between patient survival outcomes and sex.

**Table 3:**
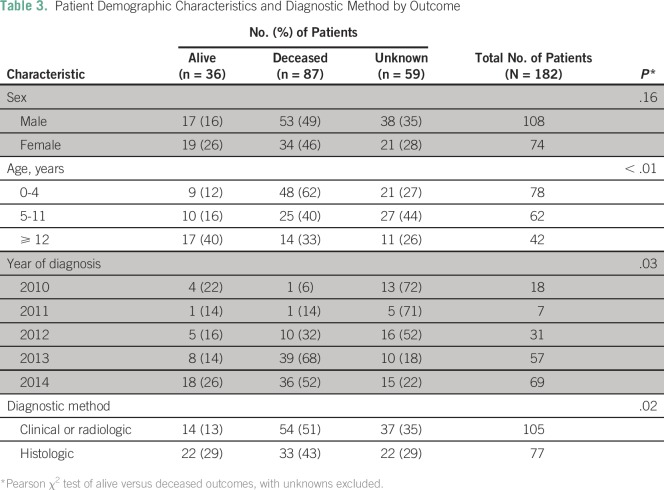
Patient Demographic Characteristics and Diagnostic Method by Outcome

### Treatment

Most patients (n = 170; 93%) received at least one chemotherapy treatment. Of the 12 patients who did not receive chemotherapy, four did not have an indication for additional therapy after surgical excision, one patient died, and seven abandoned care before chemotherapy could be initiated.

Overall, 23% of patients (n = 41) completed prescribed therapy, 40% of patients (n = 72) abandoned care, and an additional 38% (n = 69) did not abandon care but also did not complete treatment. The most commonly cited reasons for treatment incompletion in patients who did not abandon care were progressive disease (n = 34) and toxicity (n = 14; Fig 3).

## DISCUSSION

This is, to our knowledge, the first review of pediatric cancer outcomes in northern Tanzania, and it provides a better understanding of the burden of disease in this region. We report a 2-year event-free survival rate of 20% for all pediatric patients with cancer and a treatment abandonment rate of 40%. The multiple layers of complexity in the treatment of pediatric cancer in a low-resource setting are highlighted, and target areas to improve pediatric oncology outcomes in northern Tanzania, which include increased diagnostic and treatment capacities, registry and research database development, and the need for additional research to reduce treatment abandonment, are identified.

Little is known about the true incidence of pediatric cancer in Tanzania. As of 2006, only 11% of the population in sub-Saharan Africa was covered by a population-based cancer registry, and only 1% of the population was covered by a high-quality registry.^1,14^ Extrapolation of the data from this study to the available population data for the Lake Zone provides cancer incidence rates of 6.5 per million for ages 0 to 14 years and 5.8 per million for ages 0 to 19 years. However, these figures are implausibly low and are unlikely to approximate a true representation of incidence. Ribiero et al^3^ estimated the current pediatric cancer incidence in Tanzania to be 134 per million on the basis of cancer registries from neighboring countries. By using the population-based incidence rate from Ribiero et al^3^ and population data from the Tanzania National Bureau of Statistics,^15^ the projected annual incidence in children age 0 to 19 years in the Lake Zone would be 1,089. Only 298 total patient cases were identified in the 5 years of our study, which means that only 5% of all anticipated pediatric patients with cancer presented to BMC for treatment.

Although there is a deficit of identified patient cases across all cancer groups, the lack of CNS tumors and the low rate of leukemia are particularly notable in this study. According to Surveillance, Epidemiology, and End Results data, leukemia represents 27% of cancers in US children younger than 19 years of age, and CNS tumors represent 18%.^2^ This is compared with 15% and 0%, respectively, in our patient population. These discordant figures likely are due to the limited diagnostic capacity at local health centers, because the necessary diagnostic tests—such as a complete blood count or advanced imaging—typically are not available. Malignancies that present with visual masses, such as Burkitt lymphoma and Wilms tumor, are easier to recognize and are more likely to be referred to a tertiary center like BMC. This referral propensity is seen in reports across sub-Saharan Africa: visible tumors, such as Kaposi sarcoma, Burkitt lymphoma, retinoblastoma, non-Hodgkin lymphoma, and Wilms tumor, represent the most commonly reported pediatric tumors.^16^

A histopathologic diagnosis is an essential first step in cancer management, but pathology capacity is limited in Tanzania. Adesina et al^17^ reported in 2013 that there was one pathologist for every 1,877,739 people in the country. In the Lake Zone of Tanzania, BMC is the only site with pathology services, and this provides two pathologists for 15 million people. In contrast, countries with a robust pathology infrastructure, like the United States, have one pathologist for every 20,638 people.^17^ The lack of diagnostic pathology results in frequent misdiagnoses and, subsequently, inadequate, inappropriate, and often-delayed treatment.^17,18^ The importance of diagnostic pathology was confirmed in this study, as significantly better outcomes were noted in patients who received a histologic diagnosis compared with a clinical or radiographic diagnosis.

After a diagnosis is made, patients suffer from a weak clinical infrastructure. Investment in the entire spectrum of cancer infrastructure, from prevention to palliative care, should be addressed.^19^ Currently, there is only a single trained local pediatric oncologist in Dar es Salaam, and there are no pediatric oncology training programs available in Tanzania. The provision of pediatric surgical services is important for the care of many patients with cancer, but the number of pediatric surgeons in sub-Saharan Africa is low as well.^20^ As many as 50% of all patients with cancer who were identified in this study could have benefited from radiotherapy treatment, but BMC, like many other hospitals in sub-Saharan Africa, lacks radiotherapy facilities.^21,22^

The 2-year event-free survival rate in this study was 20%. Although updated overall survival rates for pediatric cancer at the national pediatric cancer treatment center in Dar es Salaam are unknown, they were similar, at 20%, before 2005.^10^ More recent diagnosis specific outcomes have shown survival rates of 33% for acute lymphoblastic leukemia^7^ and greater than 70% for Burkitt lymphoma.^11^ These data are 6% and 25%, respectively, at BMC. Since 2005, the pediatric cancer program in Dar es Salaam has had significant improvements in available resources, including relocation of the program to a pediatric hospital (Muhimbili National Hospital) that has a dedicated cancer ward; a full-time pediatric oncologist and pediatric residents; available diagnostic imaging, which includes computed tomography scans and magnetic resonance imaging; and increased treatment capacity. These differences limit direct comparisons between the two centers.^7^ Nevertheless, even when compared with published data from centers in sub-Saharan Africa that have comparable resources, survival rates at BMC remain low.^7,23,24^

One of the most distressing findings from this study was the dramatic rate of abandonment of care. These patients overcame considerable obstacles in navigation of the medical referral system, traveled to BMC, and completed the diagnostic work up; yet, three of four patients did not complete their therapy courses, and nearly half simply never came back. There is no doubt that the inability to complete proper therapy has profound implications on patient outcomes. Studies have shown that socioeconomic barriers (cost, distance, food, lodging) directly affect outcomes. For example, in patients with acute lymphoblastic leukemia in Brazil, treatment abandonment was significantly reduced after lodging, food, and transportation were provided.^25^ At Muhimbili National Hospital in Tanzania, the abandonment rate for acute leukemia was reduced to 8% after a hostel for families to stay on-site was established, which provided a strong psychosocial support for the patients.^7^ This reduction indicates that the creation of a support network may provide substantial value for BMC and other cancer hospitals in LMICs.

This study is limited in its scope to a single institution. However, BMC is the only cancer referral center in northern Tanzania, so data from this center provide the best indicator for disease burden in this region. Fewer than half of the patients receive a histologic diagnosis, so there may be some misclassifications in cancer distribution. In addition, accurate outcome data were limited by a high treatment abandonment rate. Historically, contact information was not collected as part of the medical records to provide follow-up, which may explain why almost one third of patients in this study had an unknown outcome status. If some of these patients were still alive, the true survival rate would be underestimated. However, even with an assumed underestimate, survival rates remain poor. A strong research infrastructure and clinical database would additionally reduce this ambiguity and provide more accurate outcome data.

Despite its limitations, this study makes a significant contribution to the knowledge about pediatric cancer care in limited-resource settings, and it lays the foundation for improvements to cancer care in Tanzania. This study will guide the development of a prospective clinical database that allows for more robust intervention-directed epidemiologic, treatment, and outcome studies.

In summary, current pediatric cancer outcomes in northern Tanzania are poor, and diagnostic and treatment capacities are limited. To address the burden of pediatric cancer in LMICs, such as Tanzania, there needs to be a larger trained oncologic workforce with the skills to tailor treatment specifically to children. Investments are needed in several areas, such as improved infrastructure with the necessary diagnostic equipment and supplies needed to treat these patients, as well as safety net systems to provide chemotherapies and other drugs to patients who cannot afford them. Furthermore, strategies to increase local clinical training capacity, establish quality improvement processes to track and improve mortality rates, and address barriers to access of care and factors associated with treatment abandonment are urgently needed.
